# An atypical presentation of high potassium renal secretion rate in a patient with thyrotoxic periodic paralysis: a case report

**DOI:** 10.1186/s12882-018-0971-9

**Published:** 2018-07-04

**Authors:** Mei-Lan Tu, Yu-Wei Fang, Jyh-Gang Leu, Ming-Hsien Tsai

**Affiliations:** 1 Division of Nephrology, Department of Internal Medicine, Shin-Kong Wu Ho-Su Memorial Hospital, 95, Wen-Chang Rd, Shih-Lin, Taipei, 111 Taiwan (R.O.C.); 2 Fu-Jen Catholic University School of Medicine, Taipei, Taiwan (R.O.C.)

**Keywords:** Hypokalemia, Renal potassium wasting, Thyrotoxic periodic paralysis, Hyperthyroidism, Paralysis

## Abstract

**Background:**

Hypokalemia is one of the most common clinical electrolyte imbalance problems, and thyrotoxic periodic paralysis (TPP) is a leading cause of presentation to the emergency department. Low renal potassium secretion rates, a normal acid–base balance in the blood, and hyperthyroidism are the hallmarks of suspected TPP.

**Case presentation:**

Here we report the case of a 36-year-old man who presented to the emergency department with a sudden onset of acute muscle weakness at 5 h prior to admission. Biochemistry tests revealed hypokalemia with hyperthyroidism and renal potassium wasting. TPP was initially not favored due to the presence of renal potassium wasting. However, his serum potassium level rebounded rapidly within several hours after potassium supplementation, indicating that the intracellular shifting of potassium ions was the main etiology for his hypokalemia. The early stage of TPP development may have contributed to this paradox.

**Conclusion:**

Therefore, it is premature to rule out TPP based on the presentation of high renal potassium secretion rates alone. This finding may result in an incorrect impression being made in the early stage of TTP and may consequently lead to an inappropriate potassium supplementation policy.

## Background

Thyrotoxic periodic paralysis (TPP), a disorder that primarily affects the lower extremities, occurs due to excess thyroid hormone secretion, which causes an abrupt intracellular shift of potassium ions. However, hypokalemia with hyperthyroidism does not always indicate the diagnosis of TPP. Some case reports have previously stated that patients with hyperthyroidism who presented with renal potassium wasting had a final diagnosis other than TPP, such as chronic alcoholism [1] and primary aldosteronism [2, 3]. Low renal potassium excretion, a normal acid–base balance in the blood, and hyperthyroidism are the hallmarks of TPP. However, in some conditions, TPP may present with high potassium secretion rates.

We highlight an atypical presentation of TPP with renal potassium wasting in the early stage of presentation. The early recognition of TPP is crucial to provide appropriate treatment and to avoid the risk of rebound hyperkalemia if high-dose potassium replacement is administered. Appropriate and timely management decrease morbidity, mortality, and healthcare expenditure.

## Case presentation

A 36-year-old Chinese man without any systemic medical illnesses presented to our emergency department with a 5-h history of acute general weakness that occurred upon waking up. On the day prior to symptom onset, he suffered from the feeling of low-grade fever and ate heavily before sleeping. There was no history of recent strenuous exercise or diuretic use. He denied any history of palpitations, hand tremors, abdominal pain, diarrhea, body weight loss, and numbness of limbs. Neither he nor his family members had previously experienced any such attack.

**Fig. 1:**
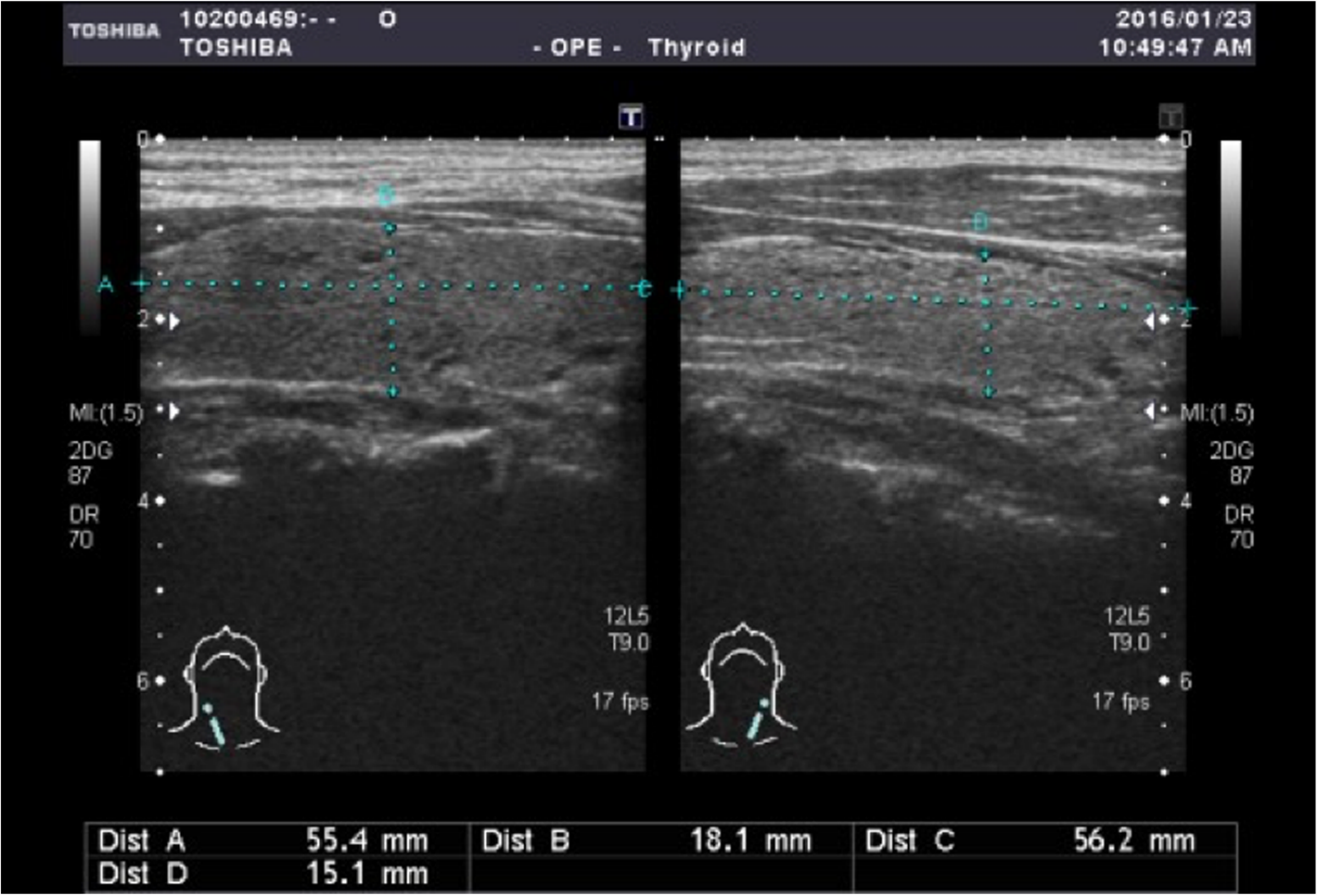
Thyroid sonogram, Right thyroid: 5.62 X 1.51 cm, Left thyroid: 5.54 X 1.81 cm

On physical examination, his blood pressure was 121/63 mmHg, body temperature was 36.6 °C, pulse rate was 102 beats per minute, and respiration rate was 18 breaths per minute. The patient had decreased muscle power especially in the lower extremities (lower limbs: proximal muscles 2/5 and distal muscles 4/5; upper limbs: proximal muscles 4/5 and distal muscles 5/5), but there was no flaccid paralysis of the lower extremities or areflexia. Other physical examination findings were unremarkable. The results of biochemical studies conducted on admission are shown in Table 1. Marked hypokalemia (2.2 mmol/L) and mild hypomagnesemia (1.8 mg/dL) were prominent findings. Urine potassium excretion indicated renal potassium wasting [transtubular potassium gradient (TTKG): 7.02; and fractional excretion of potassium (FeK): 7.12]. Potassium supplementation was initiated with potassium chloride (KCl) infusion (20 mEq of KCl in 500 mL of normal saline infused at a rate of 120 mL/h). The serum potassium level was corrected (from 2.2 to 3.8 mmol/L) within 4 h via KCl replacement (total: 19.2 mEq). The hormonal profile, including low thyroid-stimulating hormone (TSH) (< 0.0025 uIU/mL), elevated free tetraiodothyronine (T4) (1.8 ng/dL), and normal triiodothyronine (T3) (1.4 ng/mL), indicated hyperthyroidism. Because TPP was highly suspected due to the rapid resolution of hypokalemia despite high renal potassium excretion, an oral beta-blocker and anti-thyroid medication (propranolol, 10 mg BID, and methimazole, 5 mg BID) were prescribed for controlling the hyperthyroidism. Normal anti-thyroid peroxidase antibody (< 1.0 IU/mL) and high titers of thyroid-stimulating hormone receptor antibody (18.4%) indicated Graves’ disease. Thyroid sonogram revealed bilateral multinodular goiter (Fig. 1). Finally, he was discharged on day 3 of admission in a stable condition with normal serum potassium levels.

**Table 1:** Serum and urine biochemistry at admission

Parameter (reference range)	Value
Plasma	
pH (7.35–7.45)	7.37
Bicarbonate (22–26 mmol/L)	24.9
BUN (7–25 mg/dl)	13
Creatinine (0.5–1.3 mg/dl)	0.75
Na^+^ (133–145 mmol/L)	139
K^+^ (3.3–5.1 mmol/L)	2.2^a^
Cl^−^ (96–108 mmol/L)	106
Ca^++^ (3.68–5.6 mg/dl)	4.47
Phosphate (2.5–5 mg/dl)	4.9
Magnesium (1.9–2.7 mmol/L)	1.8^a^
Osmolality (278–305 mOsm/kg)	302
TSH (0.35–4.94 uIU/ml)	< 0.0025^a^
T4, Free (0.7–1.48 ng/dl)	1.8^a^
T3 (0.58–1.59 ng/ml)	1.4
Anti – TPO (0–5.61 IU/ml)	< 1.0
TSH receptor antibody	18.4%^a^
Spot urine	
pH (5–8)	6.5
Creatinine (mg/dl)	230
Na^+^ (mmol/L)	230
K^+^ (mmol/L)	48
Cl^−^ (mmol/L)	243
Osmolality (300–900 mOsm/kg)	938
TTKG (< 3)^b^	7.02
K^+^/Cr (mmol/mmol) (< 2)^b^	2.36
FeK (< 3%)^b^	7.12

^a^ Indicates abnormal values; ^b^ indicates reference range for normal renal response to hypokalemia. Abbreviation: BUN, blood urea nitrogen; TSH, thyroid stimulating hormone; TPO, thyroid peroxidase; TTKG, transtubular potassium gradient; FeK, fractional excretion of potassium

No hypokalemia was noted during the outpatient follow-up (at 9 days after discharge). Methimazole was continued to control his hyperthyroidism, and the dose was adjusted according to the thyroid function test findings. Moreover, no more muscle weakness episode occurred during the one-year follow-up in the outpatient department of endocrinology.

## Discussion

The incidence of TPP is high in Asian men from the second to fourth decades of life, and the recently increasing incidence in Western countries is due to global immigration [4]. The muscle weakness and increased risk of irregular heartbeats observed in TPP result from markedly reduced levels of potassium in the bloodstream. Increased Na^+^/K^+^-ATPase activity leads to a shift of potassium into tissues and depletes the circulation. An acid–base imbalance is generally absent in TPP. Abnormality in ion channels due to genetic mutation is thought to cause a shift of potassium into cells. The main ones are the L-type calcium channel α1-subunit [5, 6] and potassium inward rectifier 2.6 [7, 8], and this condition is therefore classified as a channelopathy [7]. Under conditions of high thyroxine levels, channelopathy leads to shifts of potassium into cells. Hyperthyroidism also increases the levels of catecholamines in the blood, increasing Na^+^/K^+^-ATPase activity [9, 10]. Increased carbohydrate intake, strenuous exercise, stress, and corticosteroids induce increased insulin levels, which activate Na^+^/K^+^-ATPase [9, 11, 12]. Additionally, male hormones increase Na^+^/K^+^-ATPase activity, and this explains why males are at a higher risk of TPP despite thyroid disease being more common in females [13–15].

**Fig. 2:**
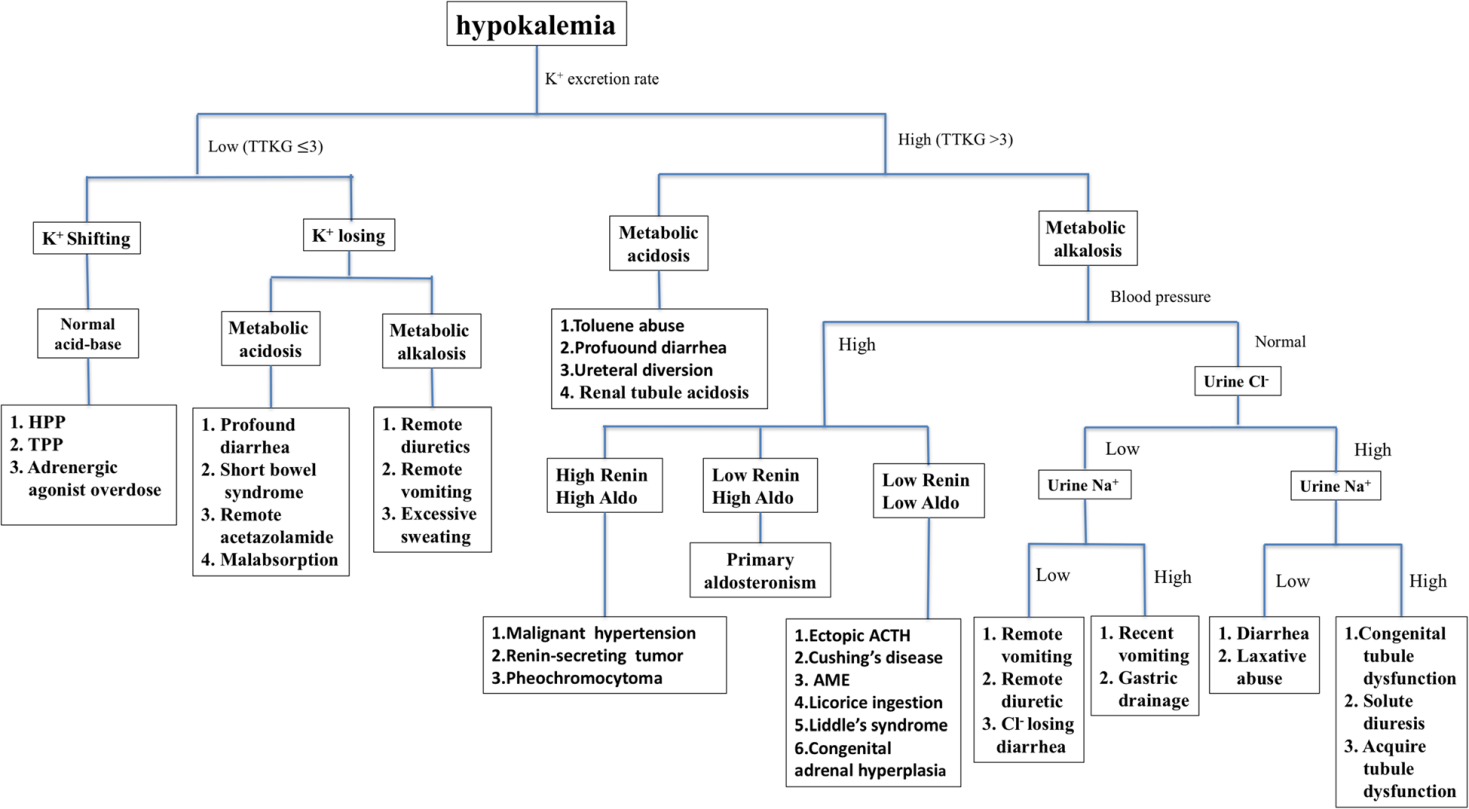
The flow chart of approaching hypokalemia [1, 2, 16–20]

The etiologies and differential diagnoses of hypokalemia are illustrated in Fig. 2. The urinary potassium excretion rate, blood acid–base status, and blood pressure are crucial to determine the cause of hypokalemic paralysis. The urinary potassium excretion rate is assessed based on TTKG (reference: 3) or spot urinary potassium–creatinine ratio (reference: 2.0 mmol/mmol). If a low urinary potassium excretion rate is noted, extrarenal loss or hypokalemic periodic paralysis should be considered. The blood acid–base status is relatively normal in TPP, whereas metabolic alkalosis or acidosis is observed in the presence of extrarenal causes, such as gastrointestinal disorders [16, 17]. If a high urinary potassium secretion rate is present, abnormal values of the blood acid–base status and blood pressure are useful clues. Coexisting metabolic acidosis suggests the presence of renal tubular acidosis, toluene use, severe diarrhea, or ureteral diversion. The presence of metabolic alkalosis with normal blood pressure advocates genetic renal tubulopathy (Gitelman syndrome and Bartter syndrome), severe diarrhea, vomiting, diuretic use, or acquired renal tubular dysfunction. The presence of metabolic alkalosis with hypertension indicates mineralocorticoid excess [1, 2, 18–20].

In the present case, the first spot urine analysis performed at the emergency department revealed a high urinary potassium excretion rate. There was no history of vomiting, diarrhea, or diuretic use. TPP was not favored despite hypokalemic paralysis with hyperthyroidism due to the presence of a high renal potassium excretion rate. However, the serially assessed serum potassium levels suddenly returned to within normal limits after several hours of potassium supplementation, indicating that the main etiology of his hypokalemia was intracellular potassium shifting. Moreover, the serum potassium level remained stable with the administration of a beta-blocker and methimazole. Low urinary phosphate excretion and hypercalciuria might further support the diagnosis of TPP. Though low urinary phosphate excretion and hypercalciuria can further support the diagnosis of TPP, we didn’t have such information. However, the other clinical features and laboratory data had contributed the highly likelihood of TPP for our case and were consistent with a diagnosis of TPP by the flow chart of hypokalemia (Fig. 2).

We developed a hypothesis to describe this paradox: the renal potassium secretion rate does not rapidly reflect an early attack of TPP. Therefore, TPP cannot be ruled out based on the detection of high renal potassium wasting in the early presentation. This finding was an additional indicator that the provisional diagnosis of TPP was not excluded. One must be alert to diagnose TPP when faced with hypokalemia and hyperthyroidism despite high renal potassium wasting. A normal blood acid–base balance may be a hint for considering such a condition.

## Conclusion

When facing a hypokalemic patient presenting with hyperthyroidism and renal potassium wasting, the impression of TPP should be taken into consideration because high renal potassium secretion may be observed in the early stage of TPP. Therefore, we should be cautious when prescribing potassium replacement to avoid rebound hyperkalemia in such a setting.

## Abbreviations

FeK: Fractional excretion of potassium
KCl: Potassium chloride
T3: Triiodothyronine
T4: Tetraiodothyronine
TPP: Thyrotoxic periodic paralysis
TSH: Thyroid-stimulating hormone
TTKG: Transtubular potassium gradient
